# Employed but Unpaid, Volunteers or Paradoxical Surplus? Sierra Leone's Unsalaried Health Workforce

**DOI:** 10.1002/hpm.70016

**Published:** 2025-08-08

**Authors:** Pieternella Pieterse, Federico Saracini

**Affiliations:** ^1^ Royal College of Surgeons in Ireland Dublin Ireland

**Keywords:** auxiliary cadre, health workers, primary care, Sierra Leone, skilled health worker ratio, universal health coverage, unsalaried

## Abstract

**Background:**

In 2016, 36.5% of Sierra Leone's health workforce consisted of unsalaried clinical staff whose payroll inclusion was deferred. The Ministry of Health introduced policies to reduce this percentage, renewing pledges to introduce health workforce planning. This paper focuses on how many unsalaried clinical staff currently work in public health facilities, based on a survey among Sierra Leone's District Health Management Teams. The study also draws on qualitative responses from unsalaried health workers regarding their coping strategies.

**Methods:**

A mixed methods approach was used, and this paper reports primarily on the survey conducted among all 16 district health authorities in 2023 and 2024. Findings from qualitative data collected among health workers, salaried and unsalaried, is also reported on.

**Findings:**

10 out of 16 districts shared staffing data, representing 55% of the population. Just over half of all Peripheral Health Unit clinical staff was unsalaried, and in 7 out of 10 districts those who were unsalaried outnumbered salaried staff. Only the capital Freetown had a large cohort of salaried clinical health workers, 58% in total. The coping strategy information from unsalaried health workers confirmed their financial hardship and formal, and sometimes informal, income generating activities.

**Discussion/Conclusion:**

Unsalaried clinical health worker numbers have increased in PHUs since 2016; an estimated 4000–5000 unsalaried clinical staff is in precarious employment, awaiting payroll inclusion. The majority of this ‘paradoxical surplus’ of health workers is trained to auxiliary cadre, meaning their eventual payroll inclusion will not increase the country's skilled‐health‐worker‐to‐population ratio, or improve Universal Health Coverage rates.

## Highlights

- Sierra Leone's unsalaried health workers have increased in number since 2016
- Up to 5000 unsalaried clinical health workers are awaiting payroll inclusion
- They rely on formal and informal income, driving up out‐of‐pocket health expenses
- Those awaiting payroll inclusion are predominantly female auxiliary health workers

## 1 Introduction

Debates regarding health systems' optimal number and type of health workers are as old as the concept of ‘health systems’ itself. Greater access to healthcare through the training and deployment of health workers has contributed to a worldwide increase in life expectancy and reduced mortality [1]. However, health worker shortages continue to exist, defined by a range of different standards [2, 3]. In recent years, as high and middle‐income country policy makers are becoming aware of ageing populations requiring more care than people used to, and having smaller populations with fewer people choosing to train as health workers, the issue has received greater attention [4, 5, 6]. Discussions regarding solutions often focus on how to train more medical personnel in countries where shortages are urgent, while technological fixes such as telemedicine are in focus since the COVID‐19 pandemic [7]. Recent studies suggest that in Sub‐Saharan Africa, a ‘paradoxical surplus’ of health workers exists [2, 8]. The problem there is the opposite; many people are trained as health workers, and the demand for them is high, but countries do not have the budget, or fiscal space, to offer paid employment to all the health workers they need. It is evident, but not always clearly understood, that large numbers of unemployed or underemployed health workers have come to exist in contexts where simultaneously skilled‐health‐worker‐to‐population‐ratio are low, and health outcomes poor [9, 10].

Sierra Leone is a compelling example of health worker unemployment [11] and the unusual way in which this phenomenon is handled at national level. In 2016 it emerged that 36.5% of all clinical healthcare staff were working without a salary [12]. These workers were not on the payroll and not counted as public sector employees in official tallies, despite being allocated posts in public primary healthcare facilities and hospitals. Such workers were therefore technically not employed; they were, what the International Labour Organisation refers to as ‘precariously employed’ [13] and were referred to as volunteers [12]. At the same time, Sierra Leone had a very low health‐worker‐to‐population‐ratio and high rates of under‐five and maternal mortality [14]. There has been no independently verified count of health workers since 2016, and this paper provides an updated estimate on their numbers, based on PHU staff data from 10 districts.

This study of Sierra Leone provides insights into how the phenomenon of paradoxical surpluses of health workers can develop; it explores the hidden un‐ and under‐employment of health workers, which is common and complex in Sub‐Saharan Africa, but not well known [15]. The purpose of this paper is to highlight the current status of unsalaried health workers in Sierra Leone, demonstrating how their numbers seem to have increased, despite policies and strategic planning to address the issue being adopted since 2017.

## 2 Background

Sierra Leone is a small west‐African nation. Despite remaining peaceful and relatively stable since emerging from an 11‐year‐old civil war in 2002, its health system remains weak and highly donor dependent [16]. It has struggled to recover from the 2014–2016 Ebola Viral Disease (EVD) epidemic [17] and its health outcomes remain among the worst worldwide [18]. A post‐Ebola count of healthcare personnel at all levels revealed that Sierra Leone relies on an unusually large cohort of so‐called ‘volunteer health workers’ [12]. The 2016 human resources for health (HRH) audit found that 48%, almost half of the health workforce was unsalaried, that is, not on the payroll. Half of the unsalaried health workers were qualified clinical staff (the other half were administrators, cleaners, porters, and drivers), who had been recruited to work in Sierra Leone's public sector facilities without being put on the payroll.

Sierra Leone has a three‐tier health system; primary healthcare is provided by 1363 Peripheral Health Units (PHUs) situated all around the country, providing basic primary care within 5 km to much of the population [19]. Secondary level care is provided at government‐run hospitals and some faith‐based hospitals, and tertiary level care is delivered by specialised referral facilities, found only in the capital Freetown [20]. Sierra Leone's Ministry of Health and Sanitation (now re‐named Ministry of Health, MoH, but throughout this paper the acronym MoHS will be used to reflect the correct name at the time of data collection) spends only 3% of its budget on primary healthcare [21]. Many maternal and child health, STI/HIV/AIDS and malaria prevention and control programmes are funded by external donors who, in 2019, financed over 93% of off‐budget costs for such health sector programmes [21].

### 2.1 Unsalaried Health Workers

The evolution of volunteerism/unpaid workers in the healthcare system in Sierra Leone can be viewed in distinct periods: post‐independence, post‐civil war, Ebola, and post‐Ebola. Historically, underinvestment in health has led to poor health outcomes in Sierra Leone [22]. After the 1991–2002 civil war, the recovery of the health system was focused on the physical rebuilding of health infrastructure and recruiting and training of new health workers who could serve in the health system [23]. Little attention was paid to the health sector payroll until NGOs stopped employing health workers directly and low‐ or unpaid health workers resorted to charging patients directly, which made care inaccessible to many, increasing mortality [24].

### 2.2 Free Healthcare

Worsening maternal mortality rates, lobbying from civil society and keen donor interest brought about the Free Healthcare Initiative (FHCI) in April 2010 [25]. It was to provide free care and medication for a range of common illnesses for pregnant and lactating mothers and children under five [26]. A recruitment drive was mounted to increase the health workforce, adding many so‐called ‘volunteer workers’ to the payroll, many were already working in public health facilities but were charging for their services [27]. The new health system design was heavily influenced [28] and funded by the donor community, with a view to gradually transfer financial responsibility to the Sierra Leone government [29]. Donor‐funded technical support within the MoHS initially kept the payroll updated, but accuracy lapsed over time [23]. To quickly increase the number of available health workers post‐war, UNICEF had been supporting Sierra Leone's Districts to run free training for women to become Maternal and Child Health Aides (MCHAs) in 14 sites nationwide [30]. Every 2 years, approximately 50 graduates emerged from each training facility, seeking employment. The majority were routinely assigned places of work within their districts, but were not added to the payroll; this is how volunteer health worker numbers continued to rapidly increase after 2010 [30].

### 2.3 Health Policies

In the aftermath of the 2014–2016 Ebola epidemic, Sierra Leone was in receipt of substantial donor funding for health systems strengthening. The HRH audit conducted at that time exposed the full magnitude of the unsalaried health workforce, to which the MoHS’ HRH Director publicly commented: ‘the total workforce is larger than previously understood, with considerable numbers of unsalaried workers’ [31]. Shortly thereafter, Sierra Leone updated its HRH Policy and Strategy for 2017–2021, acknowledging the issue of unsalaried workers:

There has been no progress towards establishment of a nationally coordinated pre‐service training plan since 2011. As a result, production often exceeds the government’s absorption capacity—particularly for lower cadre health workers. This has resulted in over 3,600 unsalaried clinical health workers providing patient services in government facilities despite not being part of the formal MoHS workforce.

HRH Strategy 2017–2021, p18 [32]

The 2017–2021 HRH Strategy contained a costed plan to absorb the majority of unsalaried clinical health workers, prioritising those who are ‘higher skilled workers for which staffing levels fall most significantly below BPEHS [Basic Package of Essential Health Services] staffing norms—such as SRNs, midwives, and CHOs’ [33] (2017b, p 47). The MoHS also planned to formulate a comprehensive training plan for all unsalaried health workers, based on further research [32, 33]. Despite the prominence of these plans in official policy documents, there is little evidence to suggest their implementation, except for the indefinite pause of MCHA and SECHN training, which came into effect in 2022. Subsequent health policy and strategy papers stayed silent on tackling unsalaried health worker issues, noting only their existence and citing their 2016 numbers [34, 35].

No previous research has been conducted into how unsalaried health workers in Sierra Leone cope while waiting to be added to the payroll. No, studies have looked into similar scenarios elsewhere. Sierra Leone is not the only country in the region that relies on putting trained health workers to work without adding them to the payroll. Post‐Ebola research revealed that in Liberia 44% and in Guinea 39% of the total health workforce is unsalaried, compared to 48% in Sierra Leone [17].

Financial hardship is experienced by many within Sierra Leone's public health workforce. Salaries are low, inflation is high [36] and this is common in many sub‐Saharan African countries [37]. It is therefore likely that salaried and unsalaried health workers alike are found to employ a range of coping strategies to supplement any income they receive [38]. Differences remain; unsalaried health workers have very few legitime means to obtain any income, as will be discussed, while those on the payroll may not have a generous wage, but they have a regular income.

## 3 Methodology

We examined the impact of Sierra Leone's unsalaried health workers using a mixed methods approach, predominantly focussing on qualitative data collection using one‐to‐one interviews, which were conducted in 2023 and 2024.

The objectives of the study were the following: 1. Determine the proportion of workers that are unsalaried; 2. Examine unsalaried workers' coping strategies and the impact this may have on their work; 3. Understand the views that free healthcare recipients and their families had of unsalaried workers (the findings of this study will be explored in a separate publication).

After initial assessment of data collected during the first research visit, it was deemed necessary to complement the study with the quantitative aspect highlighted in this paper, as the MoHS was unable to share the number of salaried (staff on the payroll, receiving monthly salaries) and unsalaried health workers with the researchers.

### 3.1 Quantitative Data Collection to Meet Objective 1 Proceeded as Follows

To obtain the records on the number of salaried and unsalaried workers in each of Sierra Leone's 16 districts, a data collection form (see supplementary file 1) was developed for online distribution to District Medical Officers (DMOs). The data collection sheet aimed to capture on the number of clinical staff working in PHUs and their status as salaried or unsalaried. The request for the data was accompanied by a copy of the research ethics approval document, a written explanation of the objectives of the study and the principal investigators' contact details. Each DMO was asked to share our request with the human resource officer (HRO) within the District Health Management Team (DHMT). At least four follow‐up approaches, by phone and text messages, were made to each DHMT (DMO, HRO or both) requesting their cooperation with the completion of the data collection form. Where possible, unresponsive DHMTs were visited in person. The data collection period ran from Oct 2023 to June 2024. The collected data was entered into an Excel sheet to facilitate basic data analysis and comparison across the districts.

### 3.2 Qualitative Data Collection Was Employed to Fulfil Study Objectives 2 and 3

In total, 110 health workers, 9 MoHS officials, 8 health worker training college managers or lecturers, 12 health worker students and 20 other key informants (primarily NGO and aid donor agency staff) were interviewed (interview guides are available as supplementary files 2–6). Data was collected during two research visits and focused on Port Loko and Bo Districts, and Western Area Urban. At each district, the DMO and HRO were interviewed, as were managers and students of the main health worker training facilities in each of the districts.

Sampling: Health facilities were selected based on size (including larger, medium sized, and smaller ones) and a mix of more and less remote locations. At small and medium sized facilities all available staff were interviewed, in larger PHUs half of all available staff were interviewed.

During visits to PHUs, researchers took note of the total number of health workers stationed at the facility, their gender, and the number of health workers present. Working conditions and coping mechanisms were discussed with all health worker interviewees. All community‐based interviews took place in the catchment areas of the health facilities where health worker interviews took place, which allowed for a certain level of triangulation of the data.

All interviews were audio recorded and the content was analysed using Lumivero NVivo15 software. Braun and Clarke's literature on thematic analysis was used to guide the analysis of the data [39, 40]. Inductive coding was followed by the grouping of codes, to ascertain which topics were most commonly discussed by the interviewees, and what patterns and themes could be discerned from the data that was collected.

Ethical approval for this study was obtained from the lead researcher's university and the Office of the Sierra Leone Ethics and Scientific Review Committee, MoHS (SLESRC No. 031/03/2023 and a 1‐year extension: 002/03/2024). During this research, Standards for Reporting Qualitative Research (SRQR) [41] were adhered to and the SRQR checklist is available as supplementary file 7.

## 4 Results

### 4.1 Quantitative Findings

All of Sierra Leone's 16 districts were targeted. In total, staffing data was received from 10 out of 16 districts. An overview of the total numbers of clinical health workers per district, salaried and unsalaried, present in the PHUs, can be found in Table 1.

**TABLE 1:** Clinical PHU Staffing numbers and their salaried or unsalaried status, per district^a^

Sierra Leone PHU staffing data collected from:	Total staff on payroll	Total staff not on payroll
Bo	600	633
Bonthe	109	219
Falaba	81	82
Kailahun	204	249
Karene	111	94
Koinadugu	91	114
Moyamba	197	259
Port Loko	223	209
Pujehun	175	217
Western Urban	822	596
Total	2613	2672

^a^ The following districts were asked but did not respond to our request for this data: Bombali, Kambia, Kenema, Kono, Tonkolili and Western Area Rural.

While the picture that emerges is not complete, the following can be observed from the data that 10 districts shared regarding their PHU staff: On average, about half (50.6%, range [42.0%–66.8%]) of all PHU staff deployed in Sierra Leone appear to be working without being on the payroll. In total, 44.6% of all inhabitants of Sierra Leone live in the six districts that did not share staffing data, but many share similar public healthcare provider profiles (regarding population, numbers of PHUs, etc.) [42]. Out of 10 districts that are reported on in the study, seven had more unsalaried than salaried PHU staff. Only Western Area Urban (the capital city Freetown) had a significantly larger number of salaried PHU staff. In Bonthe, the unsalaried/salaried staff ratio is 66.8% versus 33.2%, which is by far the worst predominance of unsalaried workers. In Moyamba, Koinadugu and Pujehun, more than 55% of the staff were unsalaried. Table 2 provides a breakdown per cadre for each of the 10 districts that provided data.

**TABLE 2:** PHU clinical staff per Sierra Leone District, per cadre and on the payroll or unsalaried, 2023/2024.

Sierra Leone PHU staffing data	Total staff PR	Total staff N‐PR	CHO/CHA/CHT PR	CHO/CHA/CHT N‐PR	CHO PR	CHO N‐ PR	CHA PR	CHA N‐ PR	CHT PR	CHT N‐PR	Midwives PR	Midwives N‐PR	SRN PR	SRN N‐PR	SECHNs PR	SECHNs N‐PR	MCH aides PR	MCH aides N‐ PR	Nursing aides PR	NA N‐PR	Lab tech PR	Lab tech N‐ PR	TBA PR	TBAs N‐ PR
Bo	600	633	94	74	0	0	0	0	0	0	81	21	0	0	150	251	275	285	0	2	0	0		
Bombali																								
Bonthe	109	219	0	0	4	0	8	12	11	8	6	0	0	0	33	51	47	145	0	0	0	3		
Falaba	81	82	0	0	4	0	3	4	1	17	9	1	0	0	22	5	40	54	1	0	1	1		
Kailahun	204	249	0	0	19	32	0	34	14	33	0	2	0	0	102	10	61	98	2	30	6	10		
Kambia																								
Karene	111	94	0	0	3	0	7	8	7	9	15	14	0	0	34	9	45	36	0	0	0	18		
Kenema																								
Koinadugu	91	114	0	0	3	6	4	1	0	1	7	1	1		24	4	49	91	3	0	0	3		7
Kono																								
Moyamba	197	259	0	0	17	15	15	16	0	0	6	1	1	0	30	10	127	72	1	0	0	8	1	137
Port Loko	223	209	0	0	2	0	1	4	14	15	16	6	1	0	70	27	114	157	5	0	0	0		
Pujehun	175	217	0	0	26	4	8	10	0	0	16	1	0	0	15	4	110	198	0	0	0	0		
Tonkolili																								
Western Rural																								
Western Urban	822	596	0	0	28	0	12	12	42	23	86	14	2	0	382	256	234	280	5	0	31	11		
Total	2613	2672	94	74	78	57	46	101	47	106	242	61	5	0	862	627	1102	1416	17	32	38	54	1	144
N‐PR Not on the payroll PR on the payroll CHO Community health officer CHA Community health assistant							CHT Community health technician SRN State registered nurse SECHN State enroled community health nurse* MCHA Maternal and child health aide*									NA Nursing aide* TBA Traditional birth attendant** * Auxiliary cadre ** Without formal training and qualification								

### 4.2 Qualitative Findings

Among the cohort of health workers who were interviewed for this study, all but a few recounted enduring a period in which they worked without being on the payroll. Both healthcare staff and MoHS representatives confirmed that health workers who do not work in the public sector as ‘volunteers’ for at least 2 years, are ineligible to apply for a permanent salaried public sector job, making working without pay a mandatory part of a health workers' path to a paid public sector healthcare post. Among the unsalaried health workers interviewed were a MCHA and a SECHN, both women, who had waited for 8 years to be added to the payroll [HW021, HW049].

At district level, MoHS staff admitted knowing about and understanding the hardship and coping mechanisms of the unsalaried staff. At national level, where MoHS staff appeared more removed from these issues, staff stressed the increase in the total number of salaried staff in recent years but were unable to say whether a concerted effort was made, as per the 2017–2021 HRH strategic plan, to offer all qualified unsalaried staff jobs as soon as this was possible. Some MoHS staff at national level suggested that unsalaried staff were informally added to the staffing rosters at district or hospital levels, without the knowledge or permission of the MoHS at national level. Several key informants suggested that between 4000 and 5000 volunteers exist in Sierra Leone's public health system, which would not be an unreasonable estimate if it can be assumed that SECHN and MCHA graduates between 2016 and 2022 were offered volunteer roles, and if a certain amount of attrition and payroll inclusion is subtracted from that number.

The most common response that was received to the question ‘how do you survive, financially?’, was 'by the grace of God'. However, many interviewees opened up and complained about the poverty that they endured. Table 3 outlines the thematically arranged responses regarding income sources and coping strategies for unsalaried workers. Many salaried health workers also discussed their financial hardship and the recent cost‐of‐living‐crisis, brought on by inflation rates of 47% in 2023 and 36% in 2024 [36].

**TABLE 3:** Unsalaried health worker income sources, support and coping strategies.

Type of income sources, support, coping strategy	At work/outside of work	Availability of this option to staff members
In‐charge of PHU provides food for shared meals	At work	Depends on goodwill of in‐charge and size of team at PHU, more likely to happen in small, remote PHU where unsalaried staff relieves burden of 24/4 workload
Receiving per diem and travel allowances for attending workshops or training	At work	Depends on size of facility and in‐charge how often staff can go. Some PHUs share these earnings among staff
Daily incentive and travel allowance to work on a vaccination campaign, bed net distribution, etc.	At work	Staff often recruited at hospitals or via DHMT, staff at remote locations less likely to take part
Receiving share of treatment fees, obtained from patients not fee exempted	At work	Only available at larger PHUs and hospitals, as maternal and child health posts only treat fee exempted patients
Support from spouse or other family member	Outside work sphere	Not often available, depends on personal circumstances
Trading or selling goods palm oil, rice, charcoal, water sachets, food, clothes, phone credit, kerosine, etc.	Outside work sphere	All trading requires capital investment. The opportunity to buy produce cheap and sell at higher prices depends on crops grown locally. Selling of small items only profitable in large PHUs and hospitals, not in small clinics with few patients
Selling medicines to patients	At work	An often‐available opportunity, unless in‐charge forbids it or has a monopoly on selling of medicines. It requires travel to a pharmacy/market where medications can be bought. In some facilities staff jointly operate the group‐funded purchase and supply of medicines
Receiving gifts from patients	At work	While gift‐giving is very common and culturally appropriate, especially to reward good service (e.g. births), the free will of patients to give gifts can be debatable

### 4.3 Health‐Facility Based Earnings

In some locations, in more remote PHUs, unsalaried staff mentioned that the ‘In‐charge’ of the facility provided food for the staff. There are several pathways to generating income while working on a voluntary basis. When staff have an opportunity to attend a training course or workshop, a transport allowance and per diem is usually on offer and is counted as a significant perk. In some health facilities these gains were shared among the whole team, while in other locations, the ‘In‐charge’, who decided whose turn in was to have this opportunity, would take a cut. Being deployed to support occasional extraordinary vaccination campaigns was also a good opportunity to earn much needed funds ‘for us, the Covid campaigns were important, they gave the jobs to us volunteers, I was grateful for that’ [HW032].

Larger health facilities (CHPs and CHCs) and hospitals cater also for patients outside of the free healthcare target group, and can legitimately charge for health services and medicines, which was occasionally highlighted. Some unsalaried workers explained that they received some of these earnings to sustain themselves [HW005, HW024, HW029]. Many others, when asked, seemed unclear if any charges levied to patients were legitimate. The absence of all signage that transparently communicated which patients were exempt from payment and which were not, and how much consultations for non‐fee exempted patients cost, seemed to have added to a general lack of clarity around charging.

### 4.4 Other Income Generating Opportunities

It is important to note that being unsalaried affects more female than male health workers, as only women could train to become MCHAs, and SECHNs are also more likely to be female [12]. Some interviewees reported that their spouse supported them financially, while others supported unemployed spouses, or were widowed or divorced. Most health workers, salaried and unsalaried, looked after other family members with the income they generated. They listed supporting their children, their elderly parents, jobless or school‐attending siblings, nieces and nephews.

Almost all unsalaried health workers earned additional income by buying and selling items. In certain locations, those with funds to invest bought locally produced palm oil or rice at harvest time, when it was cheap, which they either kept and resold when the price rose locally, or sent it to a city to be sold at a markup. Some health workers bought and sold charcoal in the same way. Many health workers struggled to eke out tiny margins selling food or clothes in a local market after work or during days off, or sold items or food to colleagues if they worked in larger health facilities: ‘I buy rice at the market here, and I sell it on a daily basis, I sell rice per cup’ [HW010]. Those working in hospitals sold items to patients and patients' family; cooked food, vegetables, sweets, water, phone credit, clothes, shoes, etc.

### 4.5 Selling Medicine

By far the most commonly‐discussed income earner was the selling of medicines to patients. Most PHUs only received consignments of FHCI medication, approximately once every 3 months, and certain disease‐specific commodities such as malaria test kits and medication, immunology supplies and some basic goods such as gloves and gauze. Medicines that could be prescribed and sold to patients outside of the FHCI target groups were not being supplied to PHUs at the time of the research. This led to health facilities, or individual health workers, organising their own supply of medication to sell to patients, many explained: ‘you buy a few drugs that can treat [patients] with, from which you can generate a small income that can help to sustain you’ [HW024]. Most medicines were bought from pharmacies in nearby towns, and sold piecemeal to patients by individual health workers, or from a joint supply funded by multiple staff with a facility. Sometimes this opportunity was monopolised by the In‐charge and/or several leading staff members. Many interviewees admitted knowing this practice was prohibited but explained that they engaged in this practice because patients would otherwise buy medication from local drug peddlers.

Some sparsely populated areas had neither pharmacies nor drug peddlers, in such a remote location, a health worker who explained “If a patient needs paracetamol, and I do not have any, she will have to get a motorbike taxi with her children to travel to the nearest town with a pharmacy. The travel alone costs her more than the price of the medication she needs. If I do not buy medicines to sell to my patients, they go without”. [HW074]

## 5 Discussion

### 5.1 Unsalaried Health Worker Increases

It seems clear that MoHS policy and strategic plans, formulated in 2017 to reduce unsalaried health worker numbers, have not been fully and successfully implemented. The total number of unsalaried health workers was greater than salaried health workers in the 10 district that disclosed PHU staffing details. Overall, the number of unsalaried clinical health workers seems to have increased in absolute terms and as a share of the total health workforce since the last official count in 2016 [12], from 36.5% to 50.6%, despite a policy being in place to reduce their numbers. The reduction was meant to have been accomplished by stopping districts and hospitals from enlisting new graduates; and by absorbing the unsalaried health workers who were recorded in 2016, onto the payroll.

Table 2 shows a relatively large number of unsalaried Community Health Officers, Community Health Assistants, Community Health Technicians and midwives, which were highlighted in the 2017–2021 HRH strategy as being a priority for recruitment, as these are considered higher skilled workers [32]. We have demonstrated that the total number of clinical unsalaried health workers engaged at PHU‐level went from 2694 in 2016, to 2672 in just 10 district in 2024, which means that in all 16 district (if we assume that the remaining six districts have roughly similar PHU staff‐to‐population ratio), the total number of unsalaried PHU staff is likely to be above 3800.

Sierra Leone's MoHS uses HR Information System (iHRIS) software, which can keep track the status of the health workforce regarding cadre, age, sex and place of work. The data collection for this study demonstrates that this information, regarding unsalaried health workers, is readily available at district level, however, MoHS has not disclosed whether they are also recorded on this system.

### 5.2 Universal Health Coverage Aspirations

The need for higher cadre health workers has become more acute in recent years since ‘achieving Universal Health Coverage (UHC) by 2030’ became the main health policy goal for Sierra Leone [34]. It is widely accepted that countries need a skilled‐health‐workers (SHW)‐to‐population ratio of 45 per 10,000 to achieve UHC [43]. Sierra Leone is estimated to have just 6.4 skilled health workers (SHWs), which includes only doctors, midwives and nurses, per 10,000 population [35]. This forces the MoHS to make tough choices; should they prioritise adding unsalaried auxiliary cadres staff like MCHAs, SECHNs and Nursing Assistants to the payroll? These are mainly primary healthcare workers which Sierra Leone relies so heavily on, but they do not meet minimum WHO SHW standards [14, 44, 45]. Or should MoHS focus on the inclusion of new health worker graduates and hire registered nurses, whose payroll inclusion would improve the country's poor Skilled Health Worker‐to‐population ratio, and would contribute to the country's health policy objective of achieving UHC [46]?

### 5.3 WHO Health Workforce Safeguarding List

Sierra Leone is on the ‘WHO health workforce support and safeguards list 2023’ [47] which strongly discourages recruitment of health workers from abroad, due to the included countries' extremely low SHW‐to‐population ratio [14]. Low‐Income Countries are thus protected by the WHO from having their health workers enticed out of the country by offers of better pay and conditions in high‐income countries, especially when a low availability of health workers causes the low SHW‐to‐population ratio. In Sierra Leone, the availability of medical doctors, specialist doctors and specialist nurses is low, and the WHO listing warrants their protection. However, as this study demonstrates, the availability of auxiliary nurses currently exceeds the number of paid posts that are available, which is why as many as 4000–5000 have taken up unsalaried posts in the hope of eventually being added to the payroll. They too are not courted for recruitment from abroad; perhaps because higher skilled labour is favoured, but maybe also because the WHO list acts as a deterrent. The discrepancy between overall health worker availability and number of skilled health workers on the payroll, demonstrates that the SHW‐to‐population ratio does not always reflect the availability of healthcare staff. In Sierra Leone, and in many other countries on the African continent, this ratio appears to be more closely linked to the availability of paid employment opportunities in the health sector, public and private [8]. While higher cadre staff recruitment is more commonly limited by availability, lower cadre employment opportunities are limited by the funding governments allocate to the health wage bill [48].

### 5.4 Health Workforce Planning

All of the above suggests that Sierra Leone overwhelmingly needs health workforce planning that combines dialogue on health workforce wage bill expansion and the development of health worker training courses, to ensure that the number of health workers that emerges from Sierra Leone's many training facilities does not vastly outstrip the number of available paid jobs in the public and private sector in the country. A regularly updated electronic HR data base that includes unsalaried health workers could offer greater transparency regarding this issue, and support overall HR planning and forecasting.

### 5.5 Health Worker Hardship

The qualitative research findings, while only briefly explored here (a paper that focuses in greater detail on all health worker interview data will be published separately, forthcoming), point to significant hardship being endured by unsalaried health workers. The data shows that many have little choice but to work without pay in order to eventually secure a pay‐rolled position. Their lack of remuneration shifts part of the problem to patients, who may feel (or be) compelled to compensate health workers for care that should be free [49]. They may also have to endure suboptimal care from health workers who cannot be present at all times due to other income generating activities [50], or feel unvalued in their jobs and might be stressed due to daily financial pressure [51]. The coping strategies discussed during the health worker interviewees were not exclusively practiced by unsalaried health workers. Interviewees who were on the payroll also found it hard to make ends meet. In Sierra Leone, 60% of the cost of healthcare in is derived from out‐of‐pocket expenses; thought to be a mix of formal and informal charges. Such costs are a barrier to prompt care seeking; studies suggest that ‘not having money for healthcare and medication’ is the most common reason why patients delay care seeking, with sometimes catastrophic consequences [52, 53].

## 6 Limitations

This study has several limitations; only 10 out of Sierra Leone's 16 districts responded to the request to share their PHU staffing data, which means that the findings are based on an incomplete picture of the unsalaried health worker situation. The 10 districts that did share their data represent over 55% of the population [42]. Although some district‐level hospital staffing numbers were shared during the data gathering exercise, the total number of hospitals for which data was obtained was too small to be a representative sample, and hospital staff were therefore not reported on in this paper. The latter meant that only limited comparisons could be made between this study to the 2016 HRH audit, which included hospital and PHU staffing data [12].

## 7 Conclusions

This study has demonstrated that Sierra Leone's health system continues to rely on a significant number of trained and qualified clinical health workers who are not on the payroll. Plans to put all trained unsalaried health workers on the payroll and stop recruiting additional staff without putting them on the payroll, contained in the 2017–2021 HRH Policy and Strategy, were not, or only partially implemented, and the number of unsalaried health workers in PHUs seems to have increased since [33]. The continued lack of health workforce planning has hampered the resolution of this problem and continues to do so. It is likely that as many as 4000–5000 predominantly auxiliary cadre health workers are currently working without pay, the majority do not meet WHO Skilled Health Worker standards.

Sierra Leone's health system needs all of its current health workers, especially given the countries fast population growth, from 7.1 million in 2016 to 8.6 million in 2024 [42]. However, the Sierra Leone government has not been willing or able to allocate sufficient public funds to pay for their salaries. This is not only a West African problem; it is common throughout the African continent [54]. The existence of a paradoxical surplus of health workers [8] needs more research, in order to be properly understood and quantified. Care should be taken to avoid linking Africa's health worker ‘surplus’ with shortages in high income countries and applying a one‐problem‐can‐alleviate‐the‐other logic. This study has shown that solutions designed to protect low‐income countries from brain drain are not always able to take contextual complexities into account. Country‐specific solutions, notably health workforce planning, should be the first step to address Sierra Leone's paradoxical health worker surplus. While opportunities such as employment abroad should not be categorically rejected, Sierra Leone and its aid donors should be urged to ensure that all current health workers are added to the payroll, and that unsalaried workers are first in line for training to upgrade them to cadres that are needed and meet WHO SHW standards, where possible.

## Conflicts of Interest

The authors declare no conflicts of interest.

## Supporting information


Supporting Information S1

Supporting Information S2

Supporting Information S3

Supporting Information S4

Supporting Information S5

Supporting Information S6

Supporting Information S7

## Data Availability

The data that support the findings of this study are available on request from the corresponding author. The data are not publicly available due to privacy or ethical restrictions.
