# Supplementary material for: Employed but Unpaid, Volunteers or Paradoxical Surplus? Sierra Leone's Unsalaried Health Workforce
Source: Int J Health Plann Manage. 2025 Aug 8;41(1):7–16. doi: 10.1002/hpm.70016 (PMC12794118; doi:10.1002/hpm.70016)
Supplement: Supplementary file 1 — Supporting Information S1 [file HPM-41-7-s004.docx]

**Data collection sheet, salaried and unsalaried health workers at District level:**

District:________________________________________________________

Total # of PHUs:__________________________________________________

Total # CHCs_____________________________________________________

Total # CHPs_____________________________________________________

Total # MCHPs___________________________________________________

Total # hospitals (pls name, + indicate public, private, faith-based)___________________

________________________________________________________________

________________________________________________________________

Staffing table – PHUs, whole district:

|  | On payroll | Not on payroll* |
| --- | --- | --- |
| CHO |  |  |
| CHT |  |  |
| CHA |  |  |
| Midwives (incl. SECHN/ Midwives) |  |  |
| SRN |  |  |
| SECHN |  |  |
| MCH Aides |  |  |
| Nursing Aides |  |  |
| Lab Technicians |  |  |
| Auxiliary staff (cleaners, porters |  |  |
| Other |  |  |

*any staff on ‘post-basic’ unpaid work experience year can be counted as ‘not on payroll’.

Hospital staff (per hospital)___________________________________________________________

|  | On payroll | Not on payroll |
| --- | --- | --- |
| CHO |  |  |
| CHT |  |  |
| CHA |  |  |
| Midwives (incl. SECHN/ Midwives) |  |  |
| SRN |  |  |
| SECHN |  |  |
| MCH Aides |  |  |
| Nursing Aides |  |  |
| Lab Technicians |  |  |
| Auxiliary staff (cleaners, porters |  |  |
| Other |  |  |

Hospital staff (per hospital)___________________________________________________________

|  | On payroll | Not on payroll |
| --- | --- | --- |
| CHO |  |  |
| CHT |  |  |
| CHA |  |  |
| Midwives (incl. SECHN/ Midwives) |  |  |
| SRN |  |  |
| SECHN |  |  |
| MCH Aides |  |  |
| Nursing Aides |  |  |
| Lab Technicians |  |  |
| Auxiliary staff (cleaners, porters |  |  |
| Other |  |  |
