# Supplementary material for: Employed but Unpaid, Volunteers or Paradoxical Surplus? Sierra Leone's Unsalaried Health Workforce
Source: Int J Health Plann Manage. 2025 Aug 8;41(1):7–16. doi: 10.1002/hpm.70016 (PMC12794118; doi:10.1002/hpm.70016)
Supplement: Supplementary file 2 — Supporting Information S2 [file HPM-41-7-s006.docx]

**Unsalaried health workers:**

1. Can you tell me a little bit about yourself – family, children, originally from, currently based at health facility location with [how many dependents/family members]?
2. What is your current level of training? When did you qualify, where did you study? Did you train as lower cadre HW first (when, where?)
3. When you located here for work, did you bring family with you? Are you supporting any family members back home?
4. Are you on the payroll? **[SWITCH IF ON PAYROLL]**
5. How long have you worked without a salary? Did you work elsewhere without being on payroll?
6. How many other colleagues do you have here, how many salaried/unsalaried? Do you know long have each of these colleagues worked here (salaried/unsalaried)? Were any put on payroll during your employment at this location (prev. location if recent arrival).
7. What are the benefits of current job: housing, free care for children, other benefits?
8. How many hours and days per week do you work? Do you work shifts, are there any arrangements for time off?
9. Are all your colleagues here, at the moment, are any off or away? (If so, why are they absent, for how long?)
10. Despite receiving some benefits, what are the challenges you might have to cope financially?
11. Do you work outside of healthcare facility? What type of income generating activity
12. Does any of these activities mean you have to leave work early, or not come to work some days?
13. Does working in the health facility provide opportunity to gain income? [prompt: per diems, gifts from patients, agreed fees set by PHU, asking patients to contribute, selling medication]
14. Do you ever provide medical care for people in the community privately, in their/ your home?
15. Do any of your colleagues here charge patients for care that should be free? Do patients accept they have to pay? Can they always afford to pay?
16. What is the situation with medication in this facility? How often do you receive FHC medications, do you experience stock-outs of any such meds?
17. How about cost recovery drugs? Does the facility receive any? Do you or your colleagues provide cost recovery drugs for patients by buying them yourself?
18. When did you realise that you might have to work without being on payroll? Was that before training during training, only when you started this job?
19. When do you expect to be put on payroll? Do you have any plans to leave this job if it doesn’t happen by…? Do other HCWs you know quit unsalaried health work?
