# Supplementary material for: Employed but Unpaid, Volunteers or Paradoxical Surplus? Sierra Leone's Unsalaried Health Workforce
Source: Int J Health Plann Manage. 2025 Aug 8;41(1):7–16. doi: 10.1002/hpm.70016 (PMC12794118; doi:10.1002/hpm.70016)
Supplement: Supplementary file 3 — Supporting Information S3 [file HPM-41-7-s002.docx]

**Salaried health workers:**

1. Can you tell me a little bit about yourself – family, children, originally from, currently based at health facility location with [how many dependents/family members]?
2. What is your current level of training? When did you qualify, where did you study? Did you train as lower cadre HW first (when, where?)
3. When you located here for work, did you bring family with you? Are you supporting any family members back home?
4. Are you on the payroll? **[SWITCH IF NOT ON PAYROLL]**
5. How long have you worked here? Did you work elsewhere?
6. How long have you been on payroll? Did you work without being on payroll before? How long?
7. Can you tell me how you managed to get on the payroll; what steps did you take?
8. How many other colleagues do you have here, how many salaried/unsalaried? Do you know long have each of these colleagues worked here (salaried/unsalaried)? Were any put on payroll during your employment at this location (prev. location if recent arrival).
9. What are the benefits of current job: housing, free care for children, other benefits?
10. How many hours and days per week do you work? Do you work shifts, are there any arrangements for time off?
11. Are all your colleagues here, at the moment, are any off or away? (If so, why are they absent, for how long?)
12. Despite being on payroll and receiving some benefits (if reported above), what are the challenges you might have to cope financially?
13. Does working in the health facility provide opportunity to gain additional income? [prompt: per diems, gifts from patients, agreed fees set by PHU, asking patients to contribute, selling medication, other means? – ask to explain each type of income]
14. Do you work outside of healthcare facility? What type of income generating activity? [go through each option and establish approximate income] Do you ever provide medical care for people in the community privately, in their homes, in your home (if yes, what type of patients/treatments)?
15. What is the situation with medication in this facility? How often do you receive FHC medications, do you experience stock-outs of any such meds?
16. How about cost recovery drugs? Does the facility receive any? Do you or your colleagues provide cost recovery drugs for patients by buying them yourself?
17. The colleagues who are not on payroll, how do they manage, financially? Do many of them have jobs, small business? Do you think some will leave this job if they don’t get put on payroll? Do other HCWs you know quit unsalaried health work?
