# Supplementary material for: Employed but Unpaid, Volunteers or Paradoxical Surplus? Sierra Leone's Unsalaried Health Workforce
Source: Int J Health Plann Manage. 2025 Aug 8;41(1):7–16. doi: 10.1002/hpm.70016 (PMC12794118; doi:10.1002/hpm.70016)
Supplement: Supplementary file 4 — Supporting Information S4 [file HPM-41-7-s001.docx]

**DHMT staff members**

1. Can you tell me a little bit about yourself? What is your role at the DHMT, how long have you done this job, what is your employment history (mainly DHMT or other?)
2. The research I am conducting is about healthcare workers and DHMT staff who are working, but are not on payroll. Do you have such workers within this DHMT? If so, can you tell me about them? Did you ever work without a salary too (pls expand)?
3. I understand that there are many health workers who work at the PHUs and the district hospital who are not on payroll; can you tell me about why that is?
4. Have the number of HW not on payroll increased in recent years, if yes, why do you think that is the case? [Probe: more HW available every year because more training courses, thus more graduates; or same amount graduating but more graduates willing to work unsalaried? More PHUs being built? Same amount of UHW, every year HW who are not on payroll quit, more UHW needed to fill gaps?]
5. How do you think staff who are not on payroll make a living?
6. Does the DHMT ever receive complaints about the behaviour of volunteer HWs?
7. Can you tell me about the process of recruiting graduates into unsalaried positions?
8. Do you recruit everyone who applies, everyone from the training courses nearby? Do you make an assessment to check how many posts you can fill in the PHUs? Do you have a maximum number of health workers per PHU? How many did you hire per year in 2022, 2021, 2020?
9. Do many unsalaried health workers leave their posts every year?
10. How many unsalaried health workers get put on payroll every year, as a percentage of total number hired? Does MoHS in Freetown also hire UHWs for Bo or those on payroll only?
11. I have also heard about unregistered HW; people who work in PHUs without being registered or asked by DHMT; does that happen in Bo? [if yes, estimates of how many]
12. Do you feel the healthcare provided in Bo is affected by the unsalaried health workers?
