# Supplementary material for: Employed but Unpaid, Volunteers or Paradoxical Surplus? Sierra Leone's Unsalaried Health Workforce
Source: Int J Health Plann Manage. 2025 Aug 8;41(1):7–16. doi: 10.1002/hpm.70016 (PMC12794118; doi:10.1002/hpm.70016)
Supplement: Supplementary file 5 — Supporting Information S5 [file HPM-41-7-s005.docx]

**Training facilities for nurses, MCH Aides, etc.**

1. What type of training facility is this, what type or courses are being offered, how long do these courses take?
2. Is this a government or private facility?
3. How many students do you take in, how many graduate each year, per cadre?
4. How much does it cost to study here [prices for different courses], what extra costs do students have to pay for [uniform, food, accommodation]?
5. Has your training institute changed the courses it offers in recent years?
6. Has your institution tried to offer other courses in recent years, higher cadres? If yes, why not successful, if no, why not?
7. Does your facility help graduates with getting jobs at the end of their courses? If so, what types of jobs, and are they paid?
8. Where do you expect your student end up working?
9. What percentage of graduates take unsalaried jobs at the end of their course? Those who don’t, what do they do?
10. Do students understand that it is likely they will have to take an unsalaried post after graduating?
11. How do you think graduates cope with working on unsalaried basis? Are these things discussed with the students?
12. Do you have an understanding of how graduates can get into the payroll? Do you teach students about that?
13. Do you think certain cadres have more chance of being put on payroll?
