# Supplementary material for: Employed but Unpaid, Volunteers or Paradoxical Surplus? Sierra Leone's Unsalaried Health Workforce
Source: Int J Health Plann Manage. 2025 Aug 8;41(1):7–16. doi: 10.1002/hpm.70016 (PMC12794118; doi:10.1002/hpm.70016)
Supplement: Supplementary file 6 — Supporting Information S6 [file HPM-41-7-s003.docx]

**Health worker trainee questionnaire**

1. What type or course are you following, how long do these courses take, what year are you in?
2. Is this a government or private facility?
3. How many students are in your year [clarify: how many students graduate each year]?
4. How much does it cost for you to study here, fees?
5. What extra costs do you have to pay for [uniform, food, accommodation]?
6. What is the reason for you to take this course?
7. Are you upgrading from a different cadre, and if yes, why?
8. Did you already work as health workers, if yes, how long? Is the government paying for your upgrade?
9. Does your facility help graduates with getting jobs at the end of their courses? If so, what types of jobs, and are they paid?
10. Where do you expect to work when you finish?
11. Are you aware that many graduates need to take unsalaried jobs at the end of their course?
12. When did you find out about having to work unsalaried? Was that before or after you started this course?
13. How will you cope with working on unsalaried basis?
14. If unsalaried work will be difficult, will you consider private sector, if so where [private hospital, set up private clinic?]
15. Do you have an understanding of how graduates can get into the payroll? Are you taught about that?
16. Do you think certain cadres have more chance of being put on payroll?
